# Status of Constipation and Its Association with Sarcopenia in Older Adults: A Population-Based Cohort Study

**DOI:** 10.3390/ijerph182111083

**Published:** 2021-10-21

**Authors:** Hyungchul Park, Jihye Lim, Ji Yeon Baek, Eunju Lee, Hee-Won Jung, Il-Young Jang

**Affiliations:** 1 Department of Gastroenterology, Asan Medical Center, University of Ulsan College of Medicine, Seoul 05505, Korea; walkside@gmail.com (H.P.); limbecca@hanmail.net (J.L.); 2 Department of Gastroenterology, Ilsan Paik Hospital, Inje University College of Medicine, Goyang 10380, Korea; 3 Division of Geriatrics, Department of Internal Medicine, Asan Medical Center, University of Ulsan College of Medicine, Seoul 05505, Korea; dreamcatch899@gmail.com (J.Y.B.); eunjulee@amc.seoul.kr (E.L.)

**Keywords:** aging, constipation, sarcopenia, geriatric assessment

## Abstract

(1) Background: As the clinical relevance of constipation and sarcopenia is not well studied, we aimed to investigate the association between them in older adults. (2) Methods: A cross-sectional study was conducted on 1278 community-dwelling older adults in South Korea. The Rome IV criteria were used to identify patients with clinically defined constipation, while sarcopenia was defined by the Asian Working Group for Sarcopenia consensus. The cohort was classified into three groups: no constipation, self-reported constipation only, and clinically defined constipation. (3) Results: The presence of constipation was associated with sarcopenia and slow gait speed (*p* < 0.001). After adjustment for possible covariates, the association with sarcopenia attenuated, while that for slow gait speed persisted. In terms of geriatric parameters, both groups with clinically defined and self-reported constipation had a higher burden of cognitive impairment, IADL disability, and lower QOL scores (*p* < 0.05) compared with those without constipation. (4) Conclusions: Sarcopenia and slow gait speed associated with constipation in community-dwelling older adults. Individuals with self-reported constipation symptoms alone showed comparable sarcopenic and geriatric burden to those with clinically defined constipation. Clinical suspicion for possible co-existing sarcopenia is warranted in older patients with constipation.

## 1. Introduction

Constipation is a common complaint of patients visiting hospitals. It is a clinical syndrome with varied and complex symptoms, and its diagnosis commonly relies on subjective measures [1]. The prevalence of constipation is estimated to range between 10 and 15% in North America [2], while in an Asian survey, it was reported in 15–23% and 11% of female and male respondents, respectively [3,4]. In a survey conducted in South Korea, the prevalence of self-reported constipation was 16.5%, while 9.2% of the participants were diagnosed as functional constipation by the Rome II criteria [5]. In the same study, laxative use was reported in 8.2% of the participants, suggesting a discrepancy between self-reported constipation, clinical diagnosis of functional constipation, and constipation-related medication use.

The clinical course of constipation tends to be chronic and can be accompanied by various symptoms that can adversely affect the quality of life and health [6,7,8]. With aging and a sedentary lifestyle, both reported as risk factors for constipation, the constipation can be more prominent in older populations characterized by multimorbidity and frailty. For instance, constipation was more prevalent in older patients with idiopathic Parkinson’s disease [9,10]. Also, studies have shown positive associations between constipation and chronic kidney disease [11]. However, only a few investigations have been done on the clinical characteristics of constipation in the older population, including geriatric parameters that may contribute to the spectrum of constipation. Additionally, investigation of constipation in community-dwelling participants was rarer.

Sarcopenia, defined as an age-related state of decreased muscle mass, strength, and/or physical performance, is a common geriatric syndrome associated with increased risks of adverse health outcomes including fall, functional decline, and death [12,13,14,15]. Decreased physical activity and poor nutritional status, the known contributing factors of constipation, have also been regarded as risk factors for sarcopenia in several cross-sectional and longitudinal studies [12,13,15,16]. Therefore, sarcopenia can be considered as a coexisting condition of constipation that causes unpleasant outcome in older people.

In this study, we aimed to assess the cross-sectional association between the clinical spectrums of constipation and sarcopenia in community-dwelling Korean older adults.

## 2. Materials and Methods

### 2.1. Study Population and Design

This study included 1278 individuals from the Aging Study of PyeongChang Rural Area (ASPRA) who underwent annual geriatric assessments between December 2018 and October 2019. Among them, 11 participants with missing bioimpedance data, and 1 subject with a colostomy, were excluded. Eventually, records of the remaining 1266 individuals were included for analysis. Details of the design and methods of the ASPRA have been described previously [17]. Briefly, older adults residing in PyeongChang County, Gangwon Province, Korea, located 180 km east of Seoul, were enrolled through public healthcare networks. Comprehensive geriatric assessments were performed annually. The inclusion criteria for participation in the ASPRA were (1) age ≥65 years, (2) registered in the National Healthcare Services, (3) ambulatory with or without an assistive device, (4) living at home, and (5) ability to provide informed consent. The exclusion criteria were (1) living in a nursing home, (2) hospitalized, and (3) bed-ridden or receiving nursing-home-level care at the time of enrollment. Following the original establishment of the study in 2014, the study area gradually expanded to PyeongChang County, and new participants were recruited continuously. The characteristics of participants from the initial cohort were comparable to those of the nationally representative sample of Korean rural communities, except for a higher proportion of agricultural workers and a lower proportion with formal education [18].

### 2.2. Assessments of Constipation

The constipation spectrum was assessed via clinical interview and the widely used Rome IV criteria. The interviewer was blinded to the participant’s general health status or sarcopenia status. After compiling all the geriatric assessments and the constipation questionnaire, substantial differences in geriatric health status were observed among participants with self-reported constipation who did not meet the Rome IV criteria and those without constipation. Hence, we classified the population into 3 groups, no constipation, self-reported constipation only, and Rome IV-defined constipation.

#### 2.2.1. No Constipation

Individuals with no self-reported constipation symptoms and who did not satisfy the clinical criteria were classified as the no constipation group.

#### 2.2.2. Self-Reported Constipation Only

Participants were interviewed by trained nurses on general bowel habit, including constipation history and current use of laxatives. Participants with self-reported constipation symptoms, but who did not satisfy the Rome IV criteria, were classified as the self-reported constipation only group.

#### 2.2.3. Clinically Diagnosed Constipation

Irritable bowel syndrome (IBS) and functional constipation (FC) were defined according to the Rome IV criteria. To identify subjects with FC, those with IBS were filtered out first. IBS was defined as recurrent abdominal pain occurring on average at least 1 day per week in the last 3 months, with an onset of at least 6 months before diagnosis, with ≥2 of the following characteristics: (1) related to defecation, (2) associated with a change in stool frequency, and (3) associated with a change in form (appearance) of stool. After excluding IBS patients, we defined clinically diagnosed constipation as the presence of symptoms satisfying the criteria for FC. FC was defined as the presence of ≥2 of the following features within the last 3 months, with symptom onset at least 6 months prior to diagnosis: (1) straining during >25% of defecations, (2) lumpy or hard stools (Bristol Stool Form Scale 1–2) in >25% of defecations, (3) sensation of incomplete evacuation in >25% of defecations, (4) sensation of anorectal obstruction or blockage in >25% of defecations, (5) need for manual maneuvers to facilitate >25% of defecations (e.g., digital evacuation, support of the pelvic floor), and (6) <3 spontaneous bowel movements per week (Table 1).

### 2.3. Measurement of Sarcopenia

#### 2.3.1. Muscle Mass

Bioelectrical impedance analyses (InBody 620; InBody, Seoul, Korea) were performed at frequencies of 5, 50, and 500 kHz to evaluate body composition, including total mass and lean mass [19]. All participants were asked to fast overnight, and four-limb impedance was measured at the standing position. Appendicular skeletal muscle mass (ASM) was calculated as the sum of the lean mass of both arms and legs provided from the manufacturer’s algorithm. ASM was divided by the height squared (ASM/ht^2^) to allow for comparison of muscle mass.

#### 2.3.2. Grip Strength

A spring-based dynamometer (T.K.K. 5401 Grip-D; Takei, Tokyo, Japan) was used to assess the handgrip strength (kg) of both arms [20]. Participants were asked to hold the dynamometer as tight as possible in a comfortable sitting position with the arm bent at 90° over the knee [21]. Each test was performed twice with intervals of >1 min. The maximum value from the dominant arm was used for the analysis.

#### 2.3.3. Gait Speed

To evaluate usual gait speed, participants were instructed to walk a total of 7 m on a level indoor surface, at the pace most comfortable and usual for them. The 4 m transit time was measured between the first footstep at the starting line and the first footstep at the 4 m line by trained personnel using a digital stopwatch. The 1.5 m intervals of each accelerating and decelerating section were excluded from the measurement [22]. The results were reported as gait speed (m/s) [23].

#### 2.3.4. Definition of Sarcopenia

We determined sarcopenia according to the Asian Working Group for Sarcopenia consensus algorithm [13]. Briefly, among the older adults with low handgrip strength and/or slow gait speed, those with concurrent low muscle mass were classified as sarcopenia (Table 2). Decreased muscle mass was defined as the sex-specific lowest quintile of ASM/ht^2^ [24], and the clinical relevance of this measurement has been previously reported in the ASPRA population. Decreased grip strength was defined as handgrip strength <26 kg and <18 kg for men and women, respectively [22]. Slow gait speed was defined as gait speed <1 m/s [13].

### 2.4. Other Geriatric Parameters

Geriatric parameters were assessed by trained nurses using standardized comprehensive geriatric assessments. Disability was defined as requiring assistance in performing any of the 7 activities of daily living (ADL; bathing, continence, dressing, eating, toileting, transferring, and washing face and hands) or the 10 instrumental activities of daily living (IADL; food preparation, household chores, going out a short distance, grooming, handling finances, laundry, managing own medications, shopping, transportation, and using a telephone) [25]. Cognitive dysfunction was determined as scores <24 on the Korean version of the Mini-Mental State Examination [26]. Depression was determined as scores ≥21 on the Korean version of the Center for Epidemiological Studies Depression (CES-D) scale [27]. A risk for malnutrition was defined as a Mini-Nutritional Assessment-Short Form (MNA-SF) score of ≤11 [28]. Polypharmacy was identified as the regular use of ≥5 different medications [17]. Multimorbidity was defined as the presence of ≥2 chronic diseases of hypertension, diabetes, malignancy, asthma, chronic lung disease, angina, myocardial infarction, heart failure, stroke, chronic kidney disease, and arthralgia [17].

### 2.5. Statistical Analysis

Baseline characteristics between the clinically defined constipation, self-reported constipation, and no constipation groups were analyzed by analysis of variance (ANOVA). We used Pearson’s correlation analysis to evaluate correlation between the presence of sarcopenia and that of constipation. The logistic regression analysis was used to estimate the odds ratios (ORs) and 95% confidence intervals (CIs) for the association between constipation and sarcopenia after adjusting for age, sex, depression, cognitive impairment, polypharmacy, and low education. We considered these confounding variables as possible risk factors for both constipation and sarcopenia [1]. All statistical analyses were performed using R statistical software version 4.0.0 (R Foundation for Statistical Computing, Vienna, Austria. URL https://www.R-project.org/ and two-sided *p* values <0.05 were considered statistically significant.

## 3. Results

### 3.1. Prevalence of Constipation and Associated Characteristics

**Figure 1:**
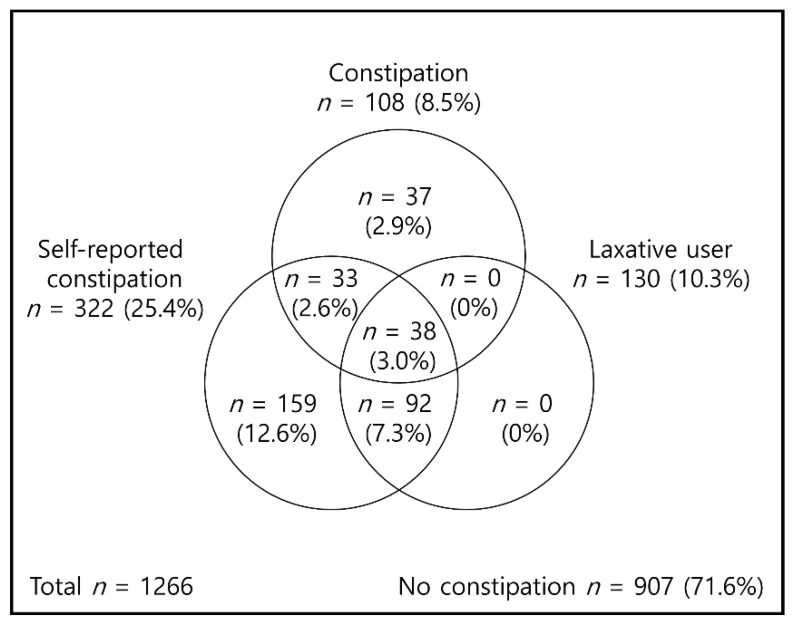
Prevalence of clinically defined constipation, self-reported constipation, and laxative use.

In the study population, the mean age was 75.3 years (SD 6.2 years), and 518 (40.9%) were male. By interview, 322 (25.4%) participants reported the presence of perceived constipation, and among them, 71 (5.6%) met the Rome IV criteria. Thirty-seven (2.9%) participants who did not self-report having constipation were diagnosed with constipation based on the Rome IV criteria. Therefore, 108 (8.5%) participants in total had clinically defined constipation, while 251 (19.9%) had self-reported constipation only (Figure 1). Prevalence of constipation is increasing according to the severity of sarcopenia: 4.9% in no sarcopenia, 6.5% in sarcopenia, and 12.6% in severe sarcopenia. Among the 130 (10.3%) individuals on laxatives, 38 (3.0%) had clinically defined constipation. 

Clinical characteristics across the constipation spectrum are shown in Table 3. Participants with constipation were generally older, less educated, had lower nutritional status, a higher burden of multimorbidity, higher rates of polypharmacy, cognitive impairment, and IADL disability, and lower QOL scores. Meanwhile, gender, the status of living alone, fall history in the previous year, and BMI did not differ significantly.

Compared with participants without constipation, those with self-reported constipation only were older, more likely to be female, living alone, less educated, had a higher burden of multimorbidity, worse mood, impaired cognitive function, and worse nutritional status, with more prevalent ADL and IADL disabilities, and lower QOL scores. Polypharmacy, BMI, and fall history in the previous year were not significantly different between the two groups. However, no significant differences in all parameters were observed between the self-reported constipation only and the clinically defined constipation groups (Table 3).

### 3.2. Association of Sarcopenia with Constipation Status

Presence of sarcopenia was correlated with constipation (rho = 0.111, *p* < 0.001). Likelihood of experiencing clinically defined constipation was higher in sarcopenic participants (OR 2.02; 95% CI 1.34–3.04, Table 4). This association attenuated after adjusting for age and sex (OR 1.36; 95% CI 0.75–2.20), and additionally for depression, cognitive impairment, polypharmacy, and low education (OR 1.17; 95% CI 0.71–1.93). Meanwhile, the significant odds for sarcopenia in self-reported constipation (OR 2.01; 95% CI 1.51–2.69) attenuated after adjusting for all possible covariates (OR 1.29; 95% CI 0.90–1.83). When clinically defined and self-reported constipation were combined as a single broad category (general constipation), the significant odds for sarcopenia (OR 2.01; 95% CI 1.56–2.59) also attenuated after adjusting for all covariates (OR 1.24; 95% CI 0.91–1.68).

### 3.3. Association of Sarcopenia Parameters with Constipation Status

Among the sarcopenia parameters (Table 5), slow gait speed was associated with clinically defined constipation (OR 1.79; 95% CI 1.37–2.35), and the association remained after adjusting for covariates (OR 1.46; 95% CI 1.10–1.94). In participants with self-reported constipation only, the significant odds for slow gait speed (OR 1.43; 95% CI 1.06–1.94) attenuated after adjusting for covariates (OR 1.19; 95% CI 0.86–1.64). Association between slow gait speed and general constipation (OR 1.79; 95% CI 1.37–2.35) remained significant even after adjusting for confounders (OR 1.46; 95% CI 1.10–1.94).

## 4. Discussion

In this population-based study on community-dwelling older adults in Korea, we found that constipation is highly prevalent, and recognized substantial discrepancies between the prevalence of self-reported and clinically defined constipation. Burdens of geriatric functional parameters were comparable between the self-reported and clinically defined constipation groups. Sarcopenia associated with the presence of constipation in general. Among the sarcopenia parameters, slow gait speed was particularly associated with constipation. To the best of our knowledge, this study is the first to address the prevalence of sarcopenia and its parameters across the spectrum of constipation in community-dwelling older adults.

In the present study, the prevalence of Rome IV-defined and self-reported constipation was 8.5% and 25.4%, respectively. Our observation is in accordance with previous studies, which reported a prevalence of 9.2% and 16.5% for functional and self-reported constipation, respectively [29], even though wide heterogeneity exists in the definitions and population characteristics involved in the studies [2].

Decreased mobility is one of the known risk factors for constipation [30], but there is still paucity in the literature on the clinical correlations between sarcopenia and constipation. Although significant correlations were observed between sarcopenia, gait speed, and constipation, we found that neither muscle mass nor grip strength associated with constipation. Even though biological mechanisms linking slow gait speed with constipation could not be further elucidated from the current study design, previous research on constipation in patients with Parkinson’s disease may provide some mechanistic insight. As a common non-motor symptom in neurodegenerative disorders characterized by dopaminergic neuron loss [10], the development of constipation is attributed to abnormal deposition of Lewy bodies in the submucosal and myenteric plexuses [31]. Studies have also shown evidence of enteric neurodegeneration involving intrinsic and extrinsic innervations as well as the interstitial cells of Cajal in idiopathic constipation [31], suggesting the possible co-existence of systemic neuromuscular aging resulting in sarcopenia and impaired mobility with enteric nerve degeneration that leads to constipation.

Sarcopenia is a well-known risk factor for adverse geriatric health outcomes and poor quality of life [12,22,32,33]. As expected, our study found that the burden of geriatric syndromes was generally worse in individuals with constipation, regardless of whether the Rome IV criteria were met. These cross-correlations between geriatric parameters, sarcopenia, and constipation may have contributed to the attenuation of significance in the multivariate logistic models. Our findings suggest the need for vigilance towards the risk of sarcopenia and geriatric syndromes in older patients with constipation.

Our study had several limitations. First, as this was performed on patients from a rural, public healthcare system, questionnaires were used and further diagnostic tests such as colonoscopy were not available. Second, given the cross-sectional design, longitudinal associations, as well as clinical outcomes associated with constipation with or without sarcopenia, could not be further assessed. Third, as our study was conducted on older adults from rural communities in South Korea, the generalizability of our results to Western populations, older adults from urban areas, and institutionalized populations is therefore unclear. Further research involving different populations and settings is hence warranted.

## 5. Conclusions

Sarcopenia and slow gait speed are associated with constipation in community-dwelling older adults. Individuals with self-reported constipation symptoms only showed comparable sarcopenic and geriatric burden to those classified with functional constipation by the Rome IV criteria. Our findings suggest that high clinical suspicion for co-existing sarcopenia is warranted in older patients with constipation.

## Figures and Tables

**Table 1:** Diagnosis of functional constipation.

**Filtering out of Irritable Bowel Syndrome**
Must fulfill the following criteria for the past 3 months:
1. Recurrent abdominal pain occurring on average at least 1 day per week
2. Pain is associated with two or more of the following criteria: a. Related to defecation b. Associated with a change in frequency of stool c. Associated with a change in form (appearance) of stool
3. Symptom onset at least 6 months prior to diagnosis
**Diagnosis of Functional Constipation**
1. Presence of ≥2 of the following features within the last 3 months: a. Straining during >25% of defecations b. Lumpy or hard stools in >25% of defecations c. Sensation of incomplete evacuation in >25% of defecations d. Sensation of anorectal obstruction or blockage in >25% of defecations e. Need for manual maneuvers to facilitate >25% of defecations f. <3 spontaneous bowel movements per week.
2. Loose stools are rarely present without the use of laxatives
3. Insufficient criteria for irritable bowel syndrome
4. Symptom onset at least 6 months prior to diagnosis

**Table 2:** Diagnosis of sarcopenia.

**Assessment of Sarcopenia Parameters**
1. Muscle strength Decreased handgrip strength < 26 kg for men and < 18 kg for women
2. Physical performance Slow gait speed <1 m/s
3. Appendicular skeletal mass (ASM) Decreased muscle mass: sex-specific lowest quintile of ASM/ht^2^
**Diagnosis of Sarcopenia**
1. Sarcopenia: Low ASM + low muscle strength OR Low physical performance
2. Severe Sarcopenia: Low ASM + low muscle strength AND Low physical performance

**Table 3:** Participant characteristics according to constipation status.

	No Constipation	Self-Reported Constipation Only	Clinically Defined Constipation	*p*-Value
	(N = 907)	(N = 251)	(N = 108)	
Age	74.7 ± 6.1	76.5 ± 6.2 ^(1)^	77.4 ± 6.5 ^(2)^	<0.001
Female	493 (54.4%)	186 (74.1%) ^(1)^	69 (63.9%)	<0.001
Living alone	219 (24.1%)	84 (33.5%) ^(1)^	27 (25.0%)	0.011
No formal education	359 (39.6%)	142 (56.6%) ^(1)^	65 (60.2%) ^(2)^	<0.001
Multimorbidity	412 (45.4%)	157 (62.5%) ^(1)^	69 (63.9%) ^(2)^	<0.001
Polypharmacy	183 (20.2%)	67 (26.7%)	32 (29.6%) ^(2)^	0.014
BMI (Kg/m^2^)	25.2 ± 3.5	25.1 ± 3.5	25.0 ± 3.7	0.648
Cognitive impairment	205 (22.7%)	88 (35.1%) ^(1)^	37 (34.3%) ^(2)^	<0.001
Depressed mood	44 (4.9%)	33 (13.1%) ^(1)^	9 (8.3%)	<0.001
Sarcopenia	415 (45.8%)	158 (62.9%) ^(1)^	68 (63.0%) ^(2)^	<0.001
Muscle mass (ASM/ht^2^)	6.6 ± 1.0	6.4 ± 2.3	6.3 ± 1.0 ^(2)^	0.003
Decreased muscle mass	285 (31.4%)	97 (38.6%)	43 (39.8%)	0.036
Grip strength (kg)	23.1 ± 9.0	19.4 ± 7.3 ^(1)^	20.4 ± 9.2 ^(2)^	<0.001
Decreased grip strength	407 (44.9%)	143 (57.0%) ^(1)^	61 (56.5%) ^(2)^	0.001
Gait speed (m/s)	1.0 ± 0.3	0.9 ± 0.3 ^(1)^	0.8 ± 0.5 ^(2)^	<0.001
Slow gait speed	497 (54.8%)	170 (67.7%) ^(1)^	83 (76.9%) ^(2)^	<0.001
Malnutrition	16 (1.8%)	10 (4.0%) ^(1)^	7 (6.5%) ^(2)^	<0.001
ADL disability	219 (24.1%)	97 (38.6%) ^(1)^	35 (32.4%)	<0.001
IADL disability	163 (18.0%)	62 (24.7%) ^(1)^	32 (29.6%) ^(2)^	0.003
Fall history	96 (10.6%)	34 (13.5%)	14 (13.0%)	0.367
EQ-5D index	0.86 ± 0.10	0.82 ± 0.12 ^(1)^	0.79 ± 0.11 ^(2)^	<0.001

ADL, activities of daily living; ASM, Appendicular skeletal muscle mass; BMI, body mass index; IADL, instrumental activities of daily living; QOL, EQ-5D index, EuroQol-5 score; ^(1)^ *p* < 0.05 between the no constipation and self-reported constipation only groups; ^(2)^ *p* < 0.05 between the no constipation and clinically defined constipation groups. No significant differences were observed between the self-reported constipation only and clinically defined constipation groups.

**Table 4:** Association of sarcopenia with constipation status.

	Model 1		Model 2		Model 3	
Variable	OR	95% CI	OR	95% CI	OR	95% CI
Self-reported constipation						
No sarcopenia	(ref)					
Sarcopenia	2.01	1.51–2.69	1.44	1.03–2.01	1.29	0.90–1.83
*p* value		<0.001		0.034		0.161
Clinically defined constipation						
No sarcopenia	(ref)					
Sarcopenia	2.02	1.34–3.04	1.36	0.75–2.20	1.17	0.71–1.93
*p* value		<0.001		0.201		0.526
General constipation						
No sarcopenia	(ref)					
Sarcopenia	2.01	1.56–2.59	1.4	1.05–1.88	1.24	0.91–1.68
*p* value		<0.001		0.024		0.169

OR, odds ratio; ref, reference; CI, confidence interval; Model 1: Unadjusted; Model 2: Model 1 with age and gender; Model 3: Model 2 with cognitive impairment, depressed mood, polypharmacy, and education.

**Table 5:** Association of sarcopenia parameters with constipation status.

	Model 1		Model 2		Model 3	
Variable	OR	95% CI	OR	95% CI	OR	95% CI
Self-reported constipation						
Normal muscle mass	(ref)					
Decreased muscle mass	1.13	0.83–1.53	1.08	0.79–1.49	1.07	0.77–1.48
*p* value		0.44		0.619		0.674
Normal grip strength	(ref)					
Decreased grip strength	1.37	1.01–1.85	1.05	0.76–1.46	1.00	0.71–1.39
*p* value		0.04		0.752		0.978
Normal gait speed	(ref)					
Slow gait speed	1.43	1.06–1.94	1.25	0.71–1.72	1.19	0.86–1.64
*p* value		0.02		0.16		0.299
Clinically defined constipation						
Normal muscle mass	(ref)					
Decreased muscle mass	1.16	0.89–1.53	1.07	0.80–1.42	1.06	0.79–1.42
*p* value		0.269		0.657		0.686
Normal grip strength	(ref)					
decreased grip strength	1.35	1.03–1.76	1.02	0.76–1.36	0.95	0.71–1.28
*p* value		0.029		0.901		0.747
Normal gait speed	(ref)					
Slow gait speed	1.79	1.37–2.35	1.54	1.16–2.04	1.46	1.10–1.94
*p* value		<0.001		0.003		0.009
General constipation						
Normal muscle mass	(ref)					
Decreased muscle mass	1.17	0.89–1.53	1.07	0.80–1.42	1.06	0.79–1.42
*p* value		0.269		0.657		0.686
Normal grip strength	(ref)					
Decreased grip strength	1.35	1.03–1.76	1.02	0.76–1.36	0.95	0.71–1.28
*p* value		0.029		0.901		0.747
Normal gait speed	(ref)					
Slow gait speed	1.79	1.37–2.35	1.54	1.16–2.04	1.46	1.10–1.94
*p* value	☐	<0.001	☐	0.003	☐	0.009

OR, odds ratio; ref, reference; CI, confidence interval; Model 1: Unadjusted; Model 2: Model 1 with age and gender; Model 3: Model 2 with cognitive impairment, depressed mood, polypharmacy, and education.

## Data Availability

The raw data of the current study are not publicly available due to the protection of participants’ personal information.
